# Transient hypercortisolism and symptomatic hyperthyroidism associated to primary hyperparathyroidism in an elderly patient: case report and literature review

**DOI:** 10.1186/1472-6823-15-4

**Published:** 2015-01-28

**Authors:** Chiara Sabbadin, Gabriella Donà, Luciana Bordin, Maurizio Iacobone, Valentina Camozzi, Caterina Mian, Decio Armanini

**Affiliations:** Department of Medicine-Endocrinology, University of Padua, Via Ospedale 105, 35128 Padua, Italy; Department of Molecular Medicine-Biological Chemistry, University of Padua, Padua, Italy; Minimally Invasive Endocrine Surgery Unit, Department of Surgery, Oncology and Gastroenterology, University of Padua, Padua, Italy

**Keywords:** Primary hyperparathyroidism, Elderly, Transient hyperthyroidism, Iodinated-contrast induced thyrotoxicosis, Functional hypercortisolism

## Abstract

**Background:**

Primary hyperparathyroidism (PHPT) is often found on routine blood tests, at a relatively asymptomatic stage. However many studies suggest different systemic effects related to PHPT, which could be enhanced by an abnormal cortisol release due to chronic stress of hyperparathyroidism. Being PHPT frequently found in the 6^th^ to 7^th^ decade of life, a careful and multifaceted approach should be taken.

**Case presentation:**

We report the case of an elderly patient with symptomatic PHPT and incidental pulmonary embolism. He was treated with hydration, zoledronic acid, cinacalcet and high-dose unfractionated heparin. Parathyroid surgery was successfully performed, but patient’s conditions suddenly worsened because of a transient thyrotoxicosis, probably induced by a previous exposure to iodine load and/or thyroid surgical manipulation. A short-term treatment with beta-blockers was introduced for symptomatic relief. The patient also presented a transient hypercortisolism with elevated ACTH, likely due to stress related not only to aging and hospitalization but also to PHPT, resolved only four months after parathyroid surgery.

**Conclusion:**

Chronic hyperparathyroidism has been linked with increased all-cause mortality. A functional chronic hypercortisolism could be established, enhancing PHPT related disorders. Only parathyroid surgery has been demonstrated to cure PHPT and complications related, showing similar outcome between older and younger patients. However, the management of post-operative period should be more careful in fragile patients. In particular, the early diagnosis and treatment of a transient post-operative thyrotoxicosis could improve recovery. Due to the increase in prevalence and the evidence of many related complications even in asymptomatic PHPT, expert opinion-based guidelines for surgical treatment of PHPT should be developed especially for elderly patients.

## Background

Primary hyperparathyroidism (PHPT) is the third most common endocrinopathy seen today, frequently found in the 6^th^ to 7^th^ decade of life. PHPT mainly is a sporadic disorder, caused in 85% of the cases by a single adenoma, in 15% by multi-gland disease and rarely by parathyroid carcinoma. Less than 10% of cases are inheritable, often associated with multi-gland hyperplasia. The most common presentation of PHPT is an asymptomatic hypercalcemia, incidentally found on routine blood tests. However, many patients may suffer from minimal symptoms such as asthenia, constipation, polyuria, hypertension and neuro-psychiatric complications [1]. In some cases a renal colic is the first presentation of the disease. In older patients, concomitant morbidity and poly-pharmacotherapy may worsen symptoms and complications, and impact the management of PHPT [2].

In this paper we report a complex case of an elderly patient with symptomatic PHPT associated with a functional transient hypercortisolism, resolved only after parathyroid surgery. The post-operative period was also characterized by a symptomatic transient thyrotoxicosis, probably induced by previous exposure to iodine load and/or thyroid surgical manipulation. The early diagnosis and treatment of this condition improved final outcome.

## Case presentation

**Figure 1:**
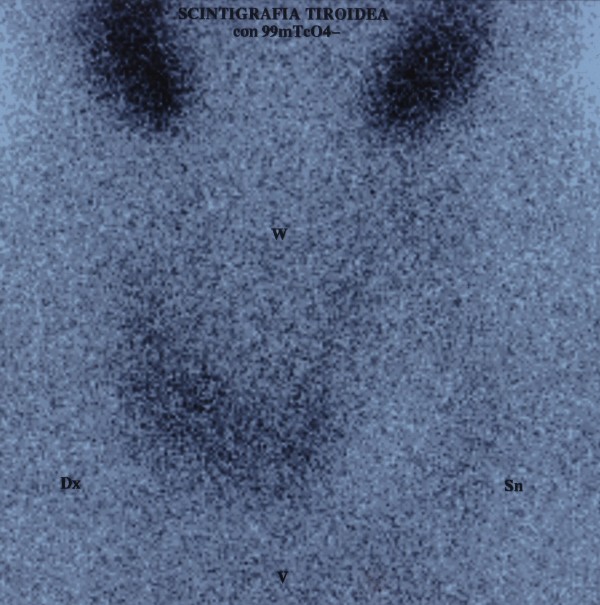
**99mTcO4 scintigraphy, performed 4 days after parathyroid surgery, evidences a reduced uptake in total thyroid tissue, especially in the lower left lobe.**

An 80-year old man was admitted to a general hospital for polyuria, vomiting, weight loss, worsening asthenia, myalgia and progressive cognitive impairment. He had a personal history of hypertension and type 2 diabetes, treated with losartan and metformin respectively. On physical examination he was sleepy, apyretic, hypertensive (upright blood pressure 150/100 mmHg) and tachycardic (100 beats for minute). He did complain of dyspnea, dry skin and mucosa and muscle weakness, without bone pain and neurological alterations. Biochemical assays revealed hypernatremia (149 mmol/L), severe hypercalcemia (4.08 mmol/L), hypophosfatemia (0.62 mmol/L), elevated levels of PTH (252 ng/L), reduced vitamin D (32 nmol/L) and slight renal failure (urea 8.7 mmol/L, creatinine 112 μmol/L). Blood count, liver and thyroid function were normal (Table 1). The electrocardiogram did not show remarkable signs of hypercalcemia. A cranial computed tomography (CT) scan excluded acute cerebrovascular events. A CT pulmonary angiography detected partial thrombosis in three segmental branches of the right upper lobe pulmonary artery. Doppler ultrasound (US) revealed a deep vein thrombosis of the left posterior tibial vein. The patient was treated with isotonic saline hydration, furosemide, supplementation of vitamin D and an injection of zoledronate 4 mg, with a mild improvement of hypercalcemia and related symptoms. Daily high-dose unfractionated heparin was also administered. The patient was then transferred to our Endocrine Unit and treated with cinacalcet, with decrease of PTH, calcemia and calciuria values. Amlodipine and insulin were also added for worsening hypertension and diabetes. Neck US revealed an enlarged thyroid with normal vascular pattern and at the lower pole of the left thyroid lobe a hypoechoic vascular nodule (14.7×10.5×9 mm), consistent with enlarged parathyroid. A sesta-MIBI scintigraphy showed a homogeneous tracer uptake over the thyroid in the early images and a remaining modest uptake in the lower left thyroid lobe in the later images; no other abnormal or ectopic uptake was found. Dual-energy x-ray absorptiometry revealed an osteopenia; all the radiological exams did not find brown tumors. Because of multiple co-morbidities, further investigations were performed to exclude other endocrine disorders: urinary metanephrine and normetanephrine values were normal, while plasma morning ACTH and daily urinary free cortisol were increased with impaired circadian cortisol rhythm, evaluated in two different measurements; serum cortisol after 1 mg overnight dexamethasone administration was not suppressed (Table 1). Direct abdomen CT was negative for adrenal diseases. Pituitary magnetic resonance imaging (MRI) evidenced a round mass, about 4 mm, without enhancement after gadolinium injection, in the left lateral portion of adenohypophysis, consistent with microadenoma. Screening for mutations of MEN genes was negative. The other pituitary hormones were normal. Because of the patient’s general conditions and a probably stress-induced hypercortisolism, no other investigations were performed, giving priority to the surgical resolution of PHPT. Bilateral neck exploration was performed with removal of the upper right parathyroid and both the lower and the upper left parathyroid gland, with quick decrease of intra-operative PTH (from 1296 to 39 ng/L). The histological diagnosis was consistent with multi-glandular hyperplasia. Laboratory tests showed a decrease of calcium and PTH levels, treated with oral calcium and calcitriol. However, four days after surgery the patient developed a sinusal tachycardia, mild heart failure and agitation alternating with stupor, without evidence of infection nor of volemic imbalance. Further investigation revealed suppressed TSH, elevated free T4 and T3 values (Table 1), with undetectable anti-thyroid and TSH receptor antibodies. Thyroid palpation was not painful. The post-operatory 99mTcO4 scintigraphy showed a reduced tracer uptake over the thyroid, especially in the lower left thyroid lobe, consistent with an inflammatory area (Figure 1). Suspecting interference by the iodinated contrast used for pulmonary angio-CT about a month before, urinary iodine excretion was measured, resulting elevated (935 μg/L, normal range 100–200). Considering a possible dual pathogenesis of thyrotoxicosis, both destructive and iodine-induced, the patient was treated with atenolol for symptomatic relief. Two months after surgery, thyroid function and ioduria were normal and beta-blocker was progressively stopped; 1 mg overnight dexamethasone test (DST) was still pathological. After other two months, calcium-phosphate balance was normal and serum PTH was near the lower limit of normal, therefore calcium and calcitriol supplementation were continued. Adrenal function was finally normalized with adequate cortisol suppression after 1 mg overnight DST (Table 1). Pituitary MRI confirmed the presence of a microadenoma, compatible with a non-secretory incidentaloma, in careful biochemical and radiological follow-up.

**Table 1:** **Principal biochemical parameters of the patient before and after surgery for primary hyperparathyroidism**

Parameter	Normal range	1 month before surgery	4 days after surgery	4 months after surgery
Ca (mmol/L)	2,1-2,5	4,2	2,5	2,2
P (mmol/L)	0,8-1,4	0,6	0,7	1,1
PTH (ng/L)	5-27	252	<4	12
ACTH (ng/L)	10-50	128	126	38
Morning salivary cortisol (ng/mL)	2,6-15,3	13,1	-	12,2
Late-night salivary cortisol (ng/mL)	0,1-5,2	15,8	-	3,4
Daily urinary cortisol (nmol/24 h)	30-193	557	281	80
Serum cortisol after 1 mg DST (nmol/L)	<50	223	-	30
TSH (mIU/L)	0,2-4	1,04	0,03	1,17
FT4 (pmol/L)	9-22	19,9	26,9	18,7
FT3 (pmol/L)	3,9-6,8	3,9	10,4	4,1

DST: dexamethasone soppression test.

## Discussion

We report an elderly patient presenting symptomatic hypercalcemia with moderate hemodynamic and neuropsychiatric failure. The detection of hypercalcemia and elevated PTH levels was diagnostic of PHPT. The biochemical/clinical presentation could also raise the suspicion of parathyroid carcinoma, which was excluded by the histological examinations. Rehydration was the first measure to take in this patient, not only to correct dehydration and improve renal failure, but also to dilute calcium excretion. Bisphosphonate administration was effective in reducing calcium levels and bone resorption, in particular recent controlled trials demonstrated the superiority of zoledronate compared with previous treatments [3]. The addition of cinacalcet may be useful in the elderly or in not-surgical candidates, being well tolerated [4]. Parathyroid surgery is the only definite cure for PHPT, but the risks and benefits of surgery should be extensively considered in the elderly, given their more fragile state and co-morbidities [5]. Preoperative imaging with ultrasonography and scintigraphy may be helpful before elective surgery and in suspected parathyroid carcinoma, even if their sensitivity drops in detecting multi-glandular disease, as happened in our case report.

Our finding of a transient thyrotoxicosis after parathyroid surgery could be due to a dual pathogenesis: a destructive thyroiditis and/or an iodine-induced hyperthyroidism. The first condition is fairly unknown and underestimated since the symptoms could be masked by other postoperative events [6]. Thyrotoxicosis seems to be related to an increased release of thyroid hormones and/or autoantigen during surgical manipulation, which could reactivate underlying autoimmune thyroiditis [7]. It could be influenced by other mechanisms, like preoperative hypercalcemic setting, pre-existing goiter and difficult parathyroid glands identification during surgical exploration [8]. Our patient had an euthyroid goiter, without abnormal MIBI-uptake in preoperative investigations nor previous or underlying autoimmune thyroiditis. Retrospectively, the only apparent risk factors were the goiter, the previous pronounced hypercalcemic condition and the occasional finding of a multi-gland disease.

The second possible cause of transient thyrotoxicosis could be related to the previous iodinated contrast media exposure, leading to hypersecretion of thyroid hormones. This phenomenon, known as the Jod-Basedow effect, usually develops over 2 to 12 weeks, typically in old patients with underlying thyroid disease or living in areas of iodine deficiency. Exposure to a large iodine load can also cause acute destructive thyroiditis in people without pre-existing thyroid disorders [9]. TcO4-scintigraphy could not discriminate the cause of hyperthyroidism, since pertechnetate is trapped by thyroid, but not organified and the resulting tracer uptake may be reduced. As happened in our case, the assessment of urinary iodine concentration may be helpful [10]. Some researchers have investigated the incidence and the role of prophylactic measures to reduce the risk of iodine-induced thyrotoxicosis, without conclusive findings [11]. In our case the concomitant neck surgery could have been a precipitating factor in the pathogenesis of hyperthyroidism.

Both these conditions are usually self-limited and anti-thyroid drugs are not indicated. However, a short-term treatment with beta-blockers could be required for symptomatic relief, especially in fragile patients.

Since chronic hyperparathyroidism has been linked with increased all-cause mortality, in asymptomatic and elderly patients the optimal timing for parathyroidectomy is controversial [12]. Cardiovascular complications are the leading cause of this increased mortality [13], linked not only to mineral homeostasis disruption but also to a direct effect of PTH on cardiovascular structures [14]. The complexity of PTH functions is further highlighted by data suggesting a bidirectional link between PTH and the renin-angiotensin-aldosterone-system, playing a synergic role in enhancing metabolic and cardiovascular complications [15]. Several studies have also evidenced an altered hypotalamic-pituitary-adrenal (HPA) axis in PHPT, potentially contributing to cortical bone damage [16]. In vitro evidence supports a stimulatory effect of PTH on cortisol secretion [17] and of calcemia on ACTH release [18]. In vivo data show a hypercortisolism, loss of circadian rhythm and lack of cortisol suppression after low-dose DST in PHPT [19], which are not always recovered after surgical cure, as happened in our case.

Alteration of cortisol expression and its circadian variability are also typical of aging, hospitalization, psychiatric and stress conditions [20]. False-positive results of the 1 mg DST could be influenced by absorption, liver or renal alterations and the use of alcohol or drugs inducing CYP3A4. Being PHPT a long-standing disease frequently affecting old patients, the activation of adrenal function seems to recall a functional hypercortisolism due to chronic stress, which could be preserved by aging and other co-morbidities, enhancing the damaging effects of prolonged exposure to stress hormones [21].

In our case, HPA axis was evaluated because of multiple co-morbidities of the patient. The data suggestive of Cushing’s syndrome were the incidental pulmonary thrombo-embolism, the uncontrolled hypertension and diabetes and the evidence of a pituitary microadenoma; however, the absence of typical stigmata and the presence of many confounding factors made the diagnosis uncertain. After discharge, further investigations excluded a Cushing’s syndrome, but the slow normalization of HPA axis only after four months seems to be related to the resolution not only of the acute stressful condition but also of PHPT.

This complex picture suggests that parathyroid surgery may improve the prognosis, normalizing also HPA axis, which could contribute to PHPT related pathologies, such as bone metabolism, psychiatric, metabolic and cardiovascular disorders [22].

## Conclusions

There is ample evidence that PHPT is associated with HPA axis alterations, which could be involved in the increased metabolic, cardiovascular and neuropsychiatric complications. A wide variety of medical therapies are available; however, only parathyroid surgery has been demonstrated to cure PHPT and complications related, showing similar outcome between older and younger patients. A transient thyrotoxicosis is a fairly underestimated condition, which could be secondary to previous iodinated contrast media exposure or to thyroid manipulation during parathyroid surgery. The early diagnosis and treatment of this complication may increase a successful recovery, especially in fragile patients.

In conclusion, due to the increase in prevalence and the evidence of many related complications even in asymptomatic PHPT, expert opinion-based guidelines for surgical treatment of PHPT should be developed especially for elderly patients.

## Consent

Written informed consent was obtained from the patient for publication of this Case report and any accompanying images. A copy of the written consent is available for review by the Editor of this journal.

## Abbreviations

PHPT: Primary hyperparathyroidism
CT: Computed tomography
US: Ultrasound
MRI: Magnetic resonance imaging
DST: Dexamethasone test
HPA: Hypotalamic-pituitary-adrenal.
